# A Reevaluation of Chitosan-Decorated Nanoparticles to Cross the Blood-Brain Barrier

**DOI:** 10.3390/membranes10090212

**Published:** 2020-08-30

**Authors:** Hernán Cortés, Sergio Alcalá-Alcalá, Isaac H. Caballero-Florán, Sergio A. Bernal-Chávez, Arturo Ávalos-Fuentes, Maykel González-Torres, Manuel González-Del Carmen, Gabriela Figueroa-González, Octavio D. Reyes-Hernández, Benjamín Floran, María L. Del Prado-Audelo, Gerardo Leyva-Gómez

**Affiliations:** 1 Laboratorio de Medicina Genómica, Departamento de Genómica, Instituto Nacional de Rehabilitación Luis Guillermo Ibarra Ibarra, Ciudad de Mexico 14389, Mexico; hcortes@inr.gob.mx; 2 Facultad de Farmacia, Universidad Autónoma del Estado de Morelos, Cuernavaca 62209, Morelos, Mexico; sergio.alcala@uaem.mx; 3 Departamento de Farmacia, Facultad de Química, Universidad Nacional Autónoma de México, Ciudad de Mexico 04510, Mexico; hiram.qfohead@gmail.com (I.H.C.-F.); q901108@hotmail.com (S.A.B.-C.); luisa.delpradoa@gmail.com (M.L.D.P.-A.); 4 Departamento de Fisiología, Biofísica y Neurociencias, Centro de Investigación y de Estudios Avanzados del Instituto Politécnico Nacional, Ciudad de Mexico 07360, Mexico; javalos22@hotmail.com (A.Á.-F.); bfloran@fisio.cinvestav.mx (B.F.); 5 CONACyT-Laboratorio de Biotecnología, Instituto Nacional de Rehabilitación Luis Guillermo Ibarra Ibarra, Ciudad de Mexico 14389, Mexico; mikegcu@gmail.com; 6 Facultad de Medicina, Universidad Veracruzana, Mendoza, Veracruz 94740, Mexico; manugonzalez@uv.mx; 7 Laboratorio de Farmacogenética, UMIEZ, Facultad de Estudios Superiores Zaragoza, Universidad Nacional Autónoma de México, Ciudad de Mexico 09230, Mexico; gabufg@comunidad.unam.mx; 8 Laboratorio de Biología Molecular del Cáncer, UMIEZ, Facultad de Estudios Superiores Zaragoza, Universidad Nacional Autónoma de México, Ciudad de Mexico 09230, Mexico; octavioreyes@comunidad.unam.mx; 9 Escuela de Ingeniería y Ciencias, Departamento de Bioingeniería, Tecnológico de Monterrey Campus Ciudad de México, Ciudad de Mexico 14380, Mexico

**Keywords:** blood-brain barrier, chitosan, nanoparticles, brain diseases, biological membranes, drug delivery systems

## Abstract

The blood-brain barrier (BBB) is a sophisticated and very selective dynamic interface composed of endothelial cells expressing enzymes, transport systems, and receptors that regulate the passage of nutrients, ions, oxygen, and other essential molecules to the brain, regulating its homeostasis. Moreover, the BBB performs a vital function in protecting the brain from pathogens and other dangerous agents in the blood circulation. Despite its crucial role, this barrier represents a difficult obstacle for the treatment of brain diseases because many therapeutic agents cannot cross it. Thus, different strategies based on nanoparticles have been explored in recent years. Concerning this, chitosan-decorated nanoparticles have demonstrated enormous potential for drug delivery across the BBB and treatment of Alzheimer’s disease, Parkinson’s disease, gliomas, cerebral ischemia, and schizophrenia. Our main objective was to highlight the high potential of chitosan adsorption to improve the penetrability through the BBB of nanoformulations for diseases of CNS. Therefore, we describe the BBB structure and function, as well as the routes of chitosan for crossing it. Moreover, we define the methods of decoration of nanoparticles with chitosan and provide numerous examples of their potential utilization in a variety of brain diseases. Lastly, we discuss future directions, mentioning the need for extensive characterization of proposed nanoformulations and clinical trials for evaluation of their efficacy.

## 1. Introduction

The brain is possibly the most sophisticated and evolved organ of human beings; thus, protection of its function is a critical issue [1]. The blood-brain barrier (BBB) is an extremely selective interface that maintains the brain homeostasis, protecting it from detrimental agents in the blood circulation and allowing the passage of glucose, amino acids, ions, hormones, oxygen, and other indispensable molecules to the brain [2]. 

The main constituents of the BBB are microvascular endothelial cells, microglia, pericytes, and astrocytes, which help to maintain its selective permeability. This feature allows the correct functioning of the synaptic and neuronal activity; thus, the BBB performs a crucial role [3]. However, brain diseases such as neurodegenerative illnesses, neuropsychiatric disorders, and brain tumors require that drugs cross the BBB to exert their therapeutic actions. Although many medications can pass the BBB by passive diffusion due to their lipophilic nature, other compounds may have difficulties to traverse it [4,5]. Many pharmacological compounds may be considered as external substances potentially harmful by the BBB, and they are impeded to cross it, degraded by enzymatic complexes, or eliminated by efflux mechanisms [6]. Due to these drawbacks, many promising therapeutic compounds potentially useful for brain diseases do not reach to be commercially available [7]. Thus, despite the essential function of the BBB, it also represents a formidable hurdle to efficient drug delivery to the brain and treatment of its diseases, making necessary the development of efficient drug delivery systems.

In this respect, diverse approaches have been proposed in recent years for the passage of drugs across the BBB, and nanoparticles have attracted significant attention for this purpose [8,9,10,11,12,13]. Particularly, chitosan-decorated nanoparticles have demonstrated strong potential for drug delivery to the brain [14]. Chitosan is a polymer natural widely utilized in different applications and approved for human use; it is a biodegradable, biocompatible, non-toxic, non-allergenic, and low-priced biomaterial; thus, chitosan is a suitable material for medical purposes [15]. Numerous studies have evaluated chitosan-coated nanosystems for the transportation of therapeutic compounds for the potential treatment of Alzheimer´s disease, Parkinson’s disease, gliomas, cerebral ischemia, and schizophrenia [16,17,18,19,20,21,22,23,24].

Our main objective was to highlight the high potential of chitosan to improve the penetrability through the BBB of nanoformulations for diseases of CNS. In this article, we describe the BBB structure and function, as well as the routes of chitosan for crossing it. Moreover, we define the methods of decoration of nanoparticles with chitosan and provide numerous examples of their potential utilization in a variety of brain diseases. Lastly, we discuss future directions, mentioning the need for extensive characterization of proposed nanoformulations and clinical trials for evaluation of their efficacy. 

## 2. Structure and Function of the Blood-Brain Barrier

**Figure 1:**
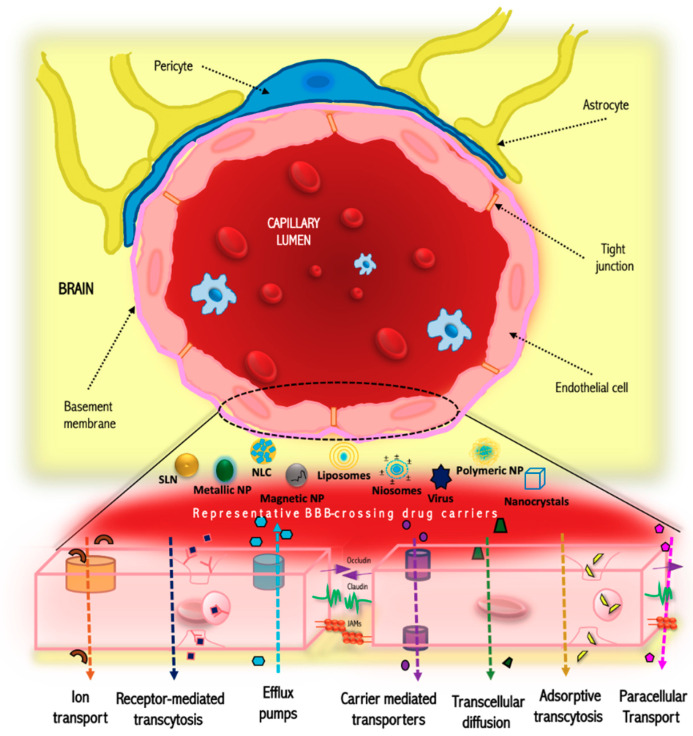
Structure and transport mechanisms of the blood-brain barrier (BBB). JAM: Junction adhesion molecules; NP: Nanoparticle; SLN: Solid lipid nanoparticle; NLC: Nanostructured lipid carriers.

The BBB is an exceptionally restrictive physical barrier that preserves the homeostasis of the brain tissue [25,26]. The surface area of the BBB is about 150 to 200 cm^2^ per gram of brain tissue, constituting a gross area of 12 to 18 m^2^ for an adult human being [2]. It is mainly composed of microvascular endothelial cells [2], which lead to the development of blood vessel walls linked by tight joints (Figure 1) [27]. On the other hand, diseases such as Alzheimer’s disease can disrupt the basement membrane, which is brought about by proteases. On the contrary, hypoxia increases the expression of laminins, which can promote vascular differentiation and BBB stability [28]; those conditions influence the barrier functions.

An essential characteristic of this barrier is the regulation of entry of drugs by severaltransportation mechanisms (Figure 1). The tight junctions restrict the passage of macromolecules or polar solutes across the endothelial and epithelial cells [29]. Concerning this, since the BBB is a lipophilic barrier, a strategy such as increasing lipophilicity, controlling the molecular weight below 400 Da, may improve its penetration through the BBB [25,27]. Proteins and peptides of high molecular weight can enter the brain through endocytotic or transcytotic mechanisms [30]. Other examples of physicochemical properties that can compromise the crossing of substances are ionization potential, pKa (ideal: low, between 7.5–10), molecular volume and flexibility (ideal: rotatable bonds fewer and in the range of less than 5), number of hydrogen bonds donors and acceptors (ideal: low, values in the range 1.5 and 2.12, respectively), partition coefficient, presence of charged system (ideal: basic compounds that in physiologic pH act as cationic moiety and interact with charged polar heads), polar surface area (ideal: low, between of 60–70) and protein binding (ideal: low, try to reduce the affinity for serum albumin) [2,31,32]. 

The BBB has shown a high selectivity that excludes more than 98% of therapeutic macromolecules and small molecules [26]. This selectivity is regulated by the presence of the P-glycoprotein, commonly referred to as the multidrug resistance protein [9]. This protein is capable of causing the drugs to return to the bloodstream through ATP-dependent efflux pumps, this event may be present even if the drug has already entered the BBB endothelium [26]. That phenomenon is frequently observed in the hydrophilic drugs or/and with high molecular weights. In this regard, there has been a significant increase in research associated with the proposal of new strategies to improve the transport of drugs through BBB in recent years. Many of these approaches are focused on the use of physical and chemical stimuli or modification of drug structure [33]. One of these strategies is the usage of nanoparticulated systems such as polymeric nanoparticles [34], solid lipid nanoparticles [35], and silica nanoparticles [36], among others. The optimal performance of nanoparticles in drug release mainly depends on the size, zeta potential of the nanoparticle, surface modifications, and biological factors such as age and sex [37]. Likewise, other studies have shown that nanoparticles with an appropriate ligand on the surface are capable of crossing the BBB. The pathway in which nanoparticles transport occurs is generally associated with receptor-mediated endocytosis, passive diffusion, liquid phase endocytosis or phagocytosis, carrier-mediated transport, or absorptive transcytosis [33,38,39]. 

## 3. Chitosan Routes for Crossing the Blood-Brain Barrier

As mentioned above, the BBB is very selective and regulates the transport of molecules into the brain, which represents a complicated impediment for delivering drugs to the brain [9,10,40,41]. In this respect, chitosan and chitosan-coated nanoparticles have been shown to enhance the efficiency of brain targeting, improving the therapeutic potential of drugs. Thus, different authors have analyzed the pathways of chitosan nanoparticles to cross the BBB, detecting several mechanisms that lead to varied hypotheses [42,43]. 

For example, in 2017, the interaction of chitosan nanoparticles and cerebral microvessel endothelial cells was evaluated [44]. The nanoparticles were decorated with antibodies recognizing human transferrin receptor (anti-TfR mAb), and the cellular uptake ability was measured in decorated and undecorated nanosystems. The cells were pre-treated with different endocytosis inhibitors (amiloride, chlorpromazine, and Mβ-cyclodextrin) to elucidate the internalization mechanism of the nanoparticles. Their results suggested that the macropinocytic pathway mediated the internalization of control nanoparticles (TfR mAb-free), meanwhile for conjugated nanoparticles, the passage was mediated by receptor-mediated transport and the macropinocytic route. Furthermore, their findings indicated that receptor-mediated transportation occurs before the aggregation of the conjugated nanoparticles due to macropinocytosis, which is known as the main cellular entry for larger particles.

On the other hand, the ability to deliver drugs into the brain by chitosan nanoparticles has been widely studied. For instance, Trapani et al. [45] evaluated the transport of dopamine-loaded chitosan nanoparticles (DA/CSNPs). For those experiments, five mg of dopamine were loaded in the nanocarrier and its internalization was assessed using the Madin–Darby canine kidney (MDCKII-MDR1) cell line. The cells were seeded in Transwell filter inserts, and the nanoparticles (previously marked with Fluorescein Isothiocyanate, FITC) were apically incubated for three h. For the control experiments, the medium without nanoparticles was employed. At different times, apical media was collected, and the FITC concentration was evaluated through fluorescence microscopy. Based on their results, the authors suggested that the internalization of the nanoparticles was mediated by an adsorptive-mediated transcytosis mechanism, being different charges between chitosan and cell monolayer the primary interaction [46,47]. 

In the same way, it was reported that Anti-neuroexcitation peptide (ANEP)-loaded N-trimethyl chitosan nanoparticles (ANEP/TMC NPs) could be transported across the BBB by absorption mediated transcytosis [48]. The results, based on fluorescence, presented a strong signal in the brain by the ANEP/TMC NPs. On the contrary, the controls exhibited a weak fluorescence. The authors attributed this behavior to the ANEP/TMC NPs positive charge (zeta potential 30.7 mV) and their interaction with the negatively charged plasma membrane on the brain capillary endothelium.

On the other hand, various authors also reported the chitosan capacity of opening the tight junctions of epithelial cells. In 2015, Kaiser et al. [49] analyzed the interaction between capsaicin-loaded chitosan-coated nanoformulations and the tight junctions of MDCK-C7 cells (an in vitro BBB model). Analysis through digital holographic microscopy revealed that chitosan opened the tight junctions. Similarly, Lien et al. [50] assessed the permeability of alkyl glyceryl chitosan nanoparticles as well as their impact on another BBB model (bEnd3 cells layer). Their results indicated that the nanoparticles induce a decrement in the electrical resistance, suggesting an effect at the tight junction level. Interestingly, the authors mentioned that not changes in the electrical resistance were observed in glial cells due to the nanoformulations, demonstrating that the variation in this property is highly specific on endothelial cells. Furthermore, the incubation of the nanoparticles in the bEnd3 cells layer stimulated the translocation of FITC-dextran through the barrier.

Altogether, these pieces of evidence indicate that chitosan and chitosan-decorated nanoparticles may cross the BBB, and therefore, these can be useful to drug delivery to the brain. Likewise, evidence suggests that the positive charge that chitosan provides to the surface of the nanoparticles could interact with the negatively charged sites on the cell membranes and tight junctions, facilitating their crossing through the BBB.

## 4. Decoration of Nanoparticles with Chitosan: Methods and Mechanisms

Attaching chitosan to the nanoparticle surface allows the improvement or addition of biological and physicochemical properties. For example, chitosan can increase or revert the nanoparticle’s zeta potential to positive, which may confer a higher biological interaction with the anionic cellular barriers and an increase in cellular internalization. Although gradually it could also produce toxicity and opsonization with blood proteins. Additionally, the decoration with chitosan enhances hydrophilicity favoring stability in aqueous environments and improving the facility to explore more administration strategies [14].

**Figure 2:**
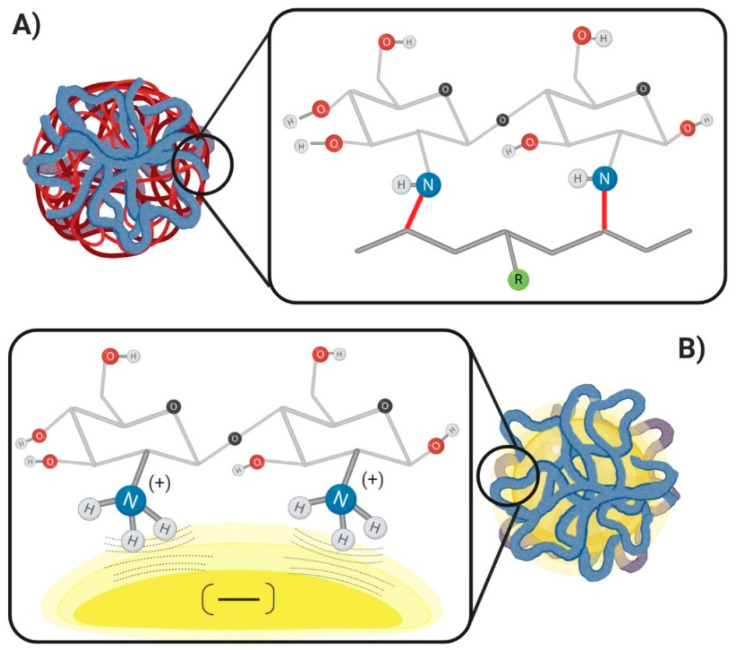
Chitosan coating mechanism. (**A**) Covalent mechanism: it is represented by the covalent bonds between the chitosan and a possible polymer (red bond). (**B**) Non-covalent mechanism: it is represented by the polar attraction forces between the cationic character on the chitosan chains and the anionic density on the nanoparticles.

The superficial modification with chitosan can be performed through the generation of new covalent bonds or by non-covalent interactions between the chemical groups present in the nanoparticle materials and chitosan molecular chains (Figure 2) [51]. The chitosan coating architecture is influenced by the method of preparation, the type of chitosan, and the molecular properties of the nanoparticle´s materials; thus, the choice of the correct mechanism will lead to better results.

Notably, the covalent mechanisms produce more stable interactions easily characterizable by analytical techniques such as infrared, nuclear magnetic resonance, or HPLC [52,53,54]. However, the procedure can become complicated, hardly scalable, and challenging to reproduce. On the other hand, the non-covalent mechanism presents weak interactions well stabilized using counterions and other polymers. The non-covalent interactions are easy to validate, scalable, and represent lower toxicity by the lack of aggressive chemical reagents or extreme conditions of processing. 

### 4.1. Non-Covalent Mechanism 

The non-covalent mechanism is based on interactions formed by coulomb attraction forces as the hydrogen bonds, at molecular and supramolecular levels. Interactions between functional groups present on the nanoparticle materials and chitosan chains lead to the stable presence of chitosan on the nanoparticle surface. These interactions are a prevalent tool to decorate the nanoparticle surface.

Generally, the process for achieving the chitosan coating on nanoparticles is through an adsorption mechanism governed by a process of charge interaction, by placing the nanoparticles in a solution of a known concentration of chitosan. However, this principle is feasible when the physical and chemical properties of the nanoparticles are stable enough, and with a predominant anionic charge once the nanoparticles are already formed. Subsequently, when the particles interact with chitosan, the positive charges in the amine groups along the chitosan backbone promote the coating over the particles. 

Nanoparticles based in polymers, lipids, protein, and inorganic materials are ideal candidates for the adsorption strategy as long as they maintain adequate stability in aqueous dispersion during the incubation process. The adsorption method may be optimized by the nanoparticle incubation in chitosan solutions with different concentrations of both reagents at distinct times until finding the ideal conditions to maintain a specific particle size, zeta potential, or degree of bioadhesion. The addition of the nanoparticles may be performed dropwise of nanoparticle solutions (to disperse the particles) or by resuspension of the particles in the chitosan solution [55,56,57,58,59,60]. The principal advantages of the non-covalent procedure are the low cost, the smooth chemical conditions, and easiness of implementation. Thus, it probably is the most explored first strategy by all the research groups for the nanoparticle’s chitosan-coating, before trying with another method.

In this regard, some authors have proposed modifications to the nanoparticle preparation methods to include the addition of chitosan during nanoparticle formation [61]. A strategy consists of adding a chitosan solution instead of pure water when the protocols needed. An example of this approach is the preparation of solid lipid nanoparticles by emulsion/evaporation/solidifying method. This protocol was modified in the last step after the solvent evaporation by Piazzini et al. [62]. The emulsion still hot is transferred to a chitosan solution (0.2%) with acetic acid (1%) at 4 °C, instead of cold water. Another modified method is the interfacial deposition of pre-formed polymers to obtain lipid core polymeric nanocapsules, where the aqueous phase contains chitosan at 0.1% [51]. 

Similarly, Mazzarino et al. [63] modified the method of nanoprecipitation-solvent displacement to obtain poly-ε-caprolactone (PCL) nanoparticles incorporating chitosan in the aqueous phase, together with the stabilizer polymer in a pH adjusted to 5. Likewise, the emulsion-solvent evaporation method to obtain polymeric nanoparticles equally can be modified to include chitosan in the aqueous phase where the emulsion is formed [17]. Interestingly, chitosan can also be adsorbed on nanoparticles by prior conjugation with other polymers, as is the case with polyethylene glycol or polyethyleneimine. In one study, iron oxide (Fe_3_O_4_) nanoparticles were manufactured by coprecipitation of ferrous and ferric chloride with ammonium hydroxide, and chitosan was adsorbed while precipitation of nanoparticles occurred [64,65,66]. With a different approach, Chung et al. explored the adsorption of chitosan in a conjugated complex with Pluronic previously activated with para-nitrophenyl chloroformate. The authors reported a first step where the chitosan is covalently bonded to the Pluronic in the presence of triethylamine. After a purification process, PLGA (poly (lactide-co-glycolide) nanoparticles were stabilized by the chitosan-pluronic in a nanoprecipitation/solvent diffusion process, to obtain a chitosan-grafted nanoparticle finally with interesting cellular uptake properties [67].

### 4.2. Covalent Mechanism 

The covalent mechanism strategies for chitosan grafted on nanoparticles surface are focused on employing chemical reactions that require mild conditions as environmental temperature, no extreme pH levels, low toxicity reagents, and an easy purification process. The chitosan crosslinking through the carbodiimide reaction is one of the most widely employed strategies. This reaction mechanism is based on the activation of carboxyl groups that produce the formation of a carbocation, then a nucleophilic attack from primary amino groups. The 1-Ethyl-3-(3-dimethyl aminopropyl) carbodiimide (EDC) is an appropriate reagent and one of the most used for this purpose. The reaction begins when EDC reacts to form the O-acylisourea intermediate product easily displaced by the amino groups in the chitosan to create the new covalent bond. EDC reaction can be performed previously, during, or after the nanoparticle synthesis [68,69,70]. Some methods apply the carboxyl activation with regents like dimethyl aminopyridine and 1,4-dioxane triethylamine, then in a second step, EDC is useful as a catalyst to achieve amide bond [71]. The chitosan addition is optimal when the carboxyl groups are still active to obtain covalent bonding. 

Transacylation reaction is another suitable approach, and it is more feasible on lipid-based nanoparticles because the ester groups present on lipidic molecules are available to react with the amino groups on the chitosan chains. The transacylation reaction occurs at 25 °C and is catalyzed through alkalization with NaOH solution, after around 15 min, the reaction is stopped by the addition of buffer solutions. The reaction produces alcohol as a secondary product, but it is easily eliminated by evaporation. Finally, the nanoparticles covered with chitosan by the new amide bond interactions are isolated and purified [72].

On the other hand, some strategies need more than one reaction to achieve the chitosan grafting by a covalent mechanism and require chemical pretreatments or conjugation with new molecules. These methods aim to conjugate the chitosan to conserve their physicochemical properties (while suffers chemical changes) and promote higher yields on the new covalent binding. For example, the previous oxidation of chitosan with KIO_4_ was required to achieve a new covalent bond between amino groups on chitosan with carboxyl groups on polyethyleneimine by reductive amination with NaBH_4_ [73]. Similarly, in another method, the chitosan needed a pretreatment based on photopolymerization reactions method for obtaining polymeric micelles. The first chitosan conjugation was with molecules like glycidyl methacrylate; this molecule reacts to form the new covalent bonding with other acrylic polymers. Subsequently, photopolymerization started with UV-radiation (1.3 mW cm^−2^) in the presence of a photoinitiator like the Irgacure D-2959, the polymerization occurred with acrylated-polymers and acrylated-chitosan to obtain chitosan conjugated nano micelles [74].

## 5. Chitosan-decorated Nanoparticles for Brain Targeting

**Figure 3:**
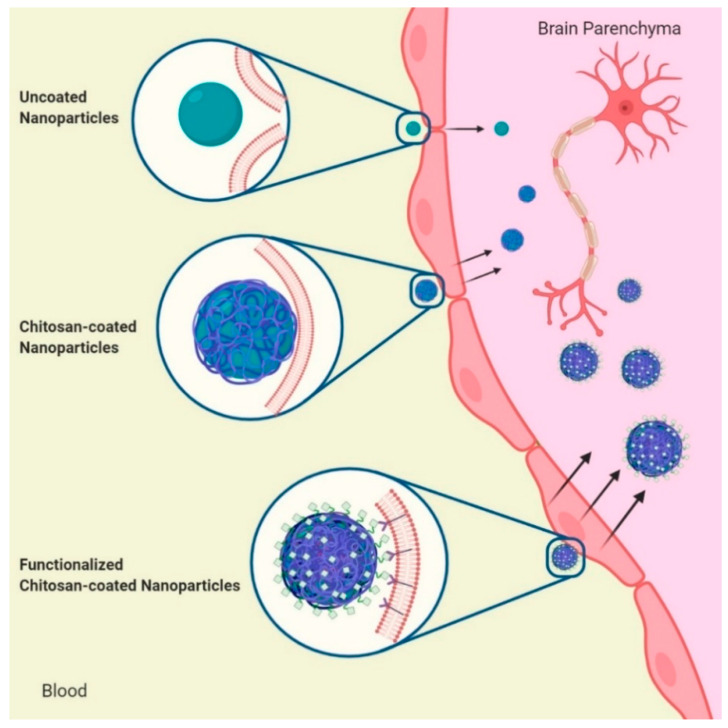
Effect of chitosan coating on nanoparticles to cross the BBB. From top to bottom. Some nanoparticles cross into the brain due to the nanometric particle size. The chitosan coating increases the interaction with the negative charge of the cell membrane in the BBB and increases the passage to the brain. The addition of specific ligands to the chitosan coating favors targeting of nanoparticles and significantly increases the crossing to the brain.

As we have seen, the positive charges of chitosan and its ability to act as a cationic polyelectrolyte offer three advantages to brain drug delivery. First, the mucoadhesive property that creates an interface of electrostatic interaction with the negative charges of the glycocalyx and the phospholipids of the epithelial membrane in the BBB [75]. Second, the enhanced permeation by the opening of tight junctions [76,77]. Third, its ability to coat surfaces and enhance the properties of other materials, such as producing positive Z potential that improves the stability of nanosystems in physiological conditions [78] (Figure 3). 

On the other hand, there are three main routes of administration to transport drugs efficiently to the brain using nanostructures, by direct injection into the brain or CNS and by intravenous pathway, which are invasives, and by inhalation (nose-to-brain pathway). The nose-to-brain route is considered the most effective method since it is non-invasive, enhances drug absorption with less systemic adverse effects, and promotes the passage through BBB, where mucoadhesion plays a determining role [79,80]. 

Nanostructures such as polymeric nanocarriers, lipid nanoparticles, micelles, and inorganic nanoparticles have been proposed as drug carriers to treat different brain diseases due to their hydrophobic properties and high affinity for membrane lipids [26,81,82]. It is noteworthy that even though some of these nanosystems have reached brain tissue more efficiently compared to chitosan nanoparticles, their ability to cross the BBB has been enhanced by coating their surface with this polycation [59,83,84,85]. 

Several works have described the use of nanoparticles decorated with chitosan as a potential alternative for brain drug targeting (see Table 1) since the available drugs to diseases of the central nervous system (CNS) have poor penetration of the BBB. This reduced penetration leads to the use of high doses, which in turn causes a wide range of side effects. Several conditions, such as gliomas, Alzheimer’s disease, Parkinson’s disease, epilepsy, meningitis, schizophrenia, migraine, and stroke, are challenging to treat due to the difficult access. Thus, it has been proposed the use of varied nature nanocarriers whose surfaces have been decorated with chitosan to promote their passage through the BBB and deliver drugs directly to the brain. Table 1 summarizes works carried out under this chitosan coating approach to achieve brain targeting; some of them are described below.

### 5.1. Polymeric Nanoparticles

Polymeric nanoparticles are the nanosystems with the highest reported usage of chitosan coating for brain delivery. For example, in several studies, chitosan-modified PLGA nanoparticles were loaded with chemotherapeutic drugs for the treatment of glioblastomas and brain cancer in in vitro and in vivo models [16,17,86]. These studies demonstrated higher cytotoxicity against malignant cells, more selectivity, and better absorption of nanoparticles by cancer cells, as well as a greater reach of brain tissue when their surfaces were modified with chitosan. The enhancing effect of chitosan was associated with a mucoadhesion linked to a longer residence time of the system in the olfactory mucosa, the electrostatic interaction with the BBB cells, the opening of tight junctions, and the control of release rate of drugs. Additionally, some nanoparticles were functionalized with sialic acid to promote the permeation through the BBB, with anti-aldehyde dehydrogenase [16] or folic acid [86] to target brain cancer stem cells.

PCL is another biodegradable polymer widely used in nanosystems development. For instance, PCL nanocapsules with a lipid core containing a statin with potential anticancer properties were proposed as possible brain drug delivery systems. The nanocarrier was coated with chitosan of different molecular weights to evaluate its effect on drug permeability in RPMI 2650 human nasal cell line. The results revealed ten times more permeation of the drug when low molecular weight chitosan was used and 6.5 times higher penetration when high molecular weight was employed, compared to uncoated nanocapsules [51]. Likewise, another work using PCL nanoparticles loaded with docetaxel to brain cancer found higher cytotoxicity with chitosan-decorated nanoparticles compared to the free docetaxel [87].

On the other hand, Alzheimer’s disease is the most prevalent neurodegenerative disease worldwide. Different studies have proposed PLGA nanoparticles to transport agents to reduce oxidative stress [18], acetyl-cholinesterase reversible inhibitors [19], and compounds that remove neurotoxic products induced by anomalous β-amyloid (Aβ) [88] for the treatment of this disease. In all cases, the chitosan coating produced an efficient passage through BBB in in vitro studies when comparing to control nanoparticles. The nanoparticles were internalized by the endocytic pathway, which appeared to be influenced by nanoparticle charges because cationic surfaces were easily attracted to endothelial cells. Likewise, in vivo studies evidenced a significant drug amount in the brain when chitosan-coated nanoparticles were administered by nasal route, which produced a retard in the degenerative progress of Aβ-insulted neurons. A different perspective of chitosan-decorated nanosystems was proposed by Jaruszewski et al. [89] for the treatment of Alzheimer’s disease. The authors tested PLGA nanoparticles loaded with 6-cumarine tested in an in vitro cerebral amyloid angiopathy model, the surface modification also included anti-amyloid antibody IgG4.1. The authors mentioned that the presence of chitosan onto nanoparticles surface provided an enhanced aqueous dispersibility and increased stability during the lyophilization process, which favored uptake at the BBB. 

Chitosan-coated PLGA nanoparticles have also been evaluated as enhancers of therapeutic molecules for the treatment of epilepsy and schizophrenia. The results demonstrated a higher amount of drug permeated to the brain when the nanoparticles were coated with the polymer. Furthermore, mucoadhesion at nasal mucosa suggested a high permanence of the nanosystem, with a release profile longer than 48 h [20,21,22]. Similar findings were observed with polymeric nanoparticles administered via intranasal to treat conditions such as cerebral ischemia (PLC nanoparticles) [23,24], for nociceptive pain (PLA (poly-lactide acid) nanoparticles) [90], or to transport neuroprotective molecules such as coenzyme Q10, or contrast agents (PLGA nanoparticles).

### 5.2. Polymeric Micelles

Polymeric micelles were proposed to CNS drug delivery to overcome the limited movement across the BBB observed in bigger nanoparticles and guarantee the retention of nanocarriers in the cerebral vasculature. Polymeric micelles are capable of solubilizing drugs of low solubility, which they have been used as drug transporters to the brain to treat glioblastomas. Pluronic P123/F68 micelles loaded with myricetin (MYR) were functionalized with chitosan to determine their anticancer activity, cellular uptake, BBB permeation, and their biodistribution in an in vivo model. The results demonstrated high cellular uptake of the drug and improved inhibitory bioactivity against tumor growth for the coated micelles. Those effects were explained by the positive charge of chitosan on the nanovehicle surface, which may have enhanced the aqueous dispersibility of MYR and increased cellular uptake [91]. 

On the other hand, another research group developed D-α-tocopheryl glycol succinate 1000-transferrin micelles conjugated covalently in amino ends of chitosan. These micelles were loaded with docetaxel with the objective of treat brain cancer. Results showed that coated micelles were 248-fold more effective after 24 h when tested on C6 glioma cells, the effect could be explained by an absorptive mediated transcytosis related to the electrostatic interaction, high mucoadhesive capacity, and a pH-sensitive response [92]. Similarly, Meenu et al. [93] designed chitosan-stearic acid micelles (stearoyl-g-glycol chitosan) bounded to the peptide TGN (a brain targeting agent). The micelles administered intravenously exhibited a better reach of brain tissue due to the surrounding hydrophilic crown caused by chitosan, which aids in the water solubility of the drug.

### 5.3. Lipid Nanoparticles

Many authors have studied solid lipid nanoparticles (SLN) as drug delivery systems to CNS due to the affinity of lipids for the BBB. However, as previously mentioned, different properties of the nanocarrier are determining factors to go through BBB, and the neutral charge of lipid nanosystems is a limitation. For this reason, SLN made of behenic acid [94] and Vit E:gelucire 44/14 [95] were decorated with chitosan to get a cationic nanocarrier and enhance the BBB permeation of paclitaxel and temozolomide, respectively. Both formulations presented increased cytotoxicity, which was explained by the increased permeability through the BBB monolayer compared to free drug, as well as an increase in viscosity and residence time of the system in the nasal region. Another report described SLN loaded with a siRNA against Aβ-site amyloid precursor protein cleaving enzyme 1, which is a critical enzyme in the development of Alzheimer’s disease. The formulation demonstrated a positive effect of chitosan coating on permeation into the brain, highlighting the impact on Z potential and siRNA stability [96].

Similarly, chitosan-decorated nanostructured lipid carriers (NLC) were devised to administrate therapeutic agents (ropinirole or glial cell-derived neurotrophic factor) to treat Parkinson’s disease. The nanocarriers were evaluated in in vivo models after a nasal administration, producing a behavioral improvement in Parkinsonian rats. The NLC containing anti-Parkinson drugs confirmed the potential of the chitosan coating to pass the BBB, with a high flux of drug during permeability studies in the nasal mucosa attributed to the opening of tight epithelial junctions [55,56,97].

Other reports described a significant difference regarding the effectiveness of SLN and NLC in brain targeting by the presence of the chitosan coating concerning non-coating ones. Therefore, these nanosystems could offer therapeutic alternatives for schizophrenia (4 fold higher brain bioavailability for asenapine) [98], migraine (improved cross through BBB and significantly anti-migraine activity, with Cmax in the brain seven times higher) [99,100], neuroprotection (against different CNS conditions by transporting peptides or molecules neuroprotectors) [101,102,103], and pain (by the use of amphiphilic lipid systems to carry analgesic peptides to the brain) [104].

### 5.4. Liposomes and Niosomes

Liposomes are considered with high potential for nose-brain delivery when they are administered in aerosol; thus, the permeation-enhancing effects and mucoadhesive properties of chitosan have been exploited by decorating their surface. For instance, liposomes loaded with levodopa or ghrelin were functionalized with chitosan for the potential treatment of Parkinson’s disease [105] and Cachexia [106], respectively. In both studies, the authors observed no changes regarding drug permeation. However, they reported a better mucoadhesion, which could extend the residence time of the formulation in the nasal cavity, thus optimizing drug transfer to the brain, producing higher therapeutic efficacy for those groups that were treated with chitosan-coated liposomes.

On the other hand, niosomes are self-assembled systems consisting mainly of nonionic surfactants with an aqueous nucleus. These systems have been evaluated in brain therapy due to their ability to improve the poor bioavailability of hydrophilic drugs that target the CNS. Its limiting factor, like some lipid nanoparticles and liposomes, is the practically neutral charge of the nanosystem. In this regard, a study was performed to encapsulate pentamidine, a drug that has shown a neuroprotective effect against Alzheimer’s disease, into niosomes prepared with Tween^®^ 20. According to the authors, the presence of chitosan on the niosomes surface was fundamental because of its penetration enhancer properties through BBB [107].

### 5.5. Inorganic Nanoparticles

Inorganic core nanoparticles have also been decorated by covalent bonding with chitosan copolymers, adding some therapeutic agents to be proposed as theragnostic systems in brain disorders. Chitosan copolymers stabilize the nanosystems by preventing particle agglomeration as a sterically stabilizing corona under physiologic pH conditions. For cancer therapy, chitosan-coated magnetic nanoparticles have been tested intravenously, subcutaneously, or directly injected to the tumor to deliver siRNAs, genes, or proteins such as O^6^-benzylguanine [64,66,108,109]. For all cases, excellent biodistribution in the brain was observed because the cationic chitosan may interact with the negatively charged brain endothelium via electrostatic interaction, triggering adsorptive-mediated transport across the BBB [110]. Remarkably, the positive charge may be used to bind proteins or DNA to the magnetic nanoparticle surface [65].

On the other hand, gold nanoparticles functionalized with brain-derived neurotrophic factor and coated with chitosan were proposed as potential perspectives in injured nerve regeneration [111]. Finally, a recent work using Magnevist^®^ core nanoparticles (MRI contrast agent) conjugated with chitosan and loaded with cyclophosphamide confirmed that the positive charge of nanosystems enhanced their transport across BBB, allowing reaching the brain tissue, generating a diagnostic image, and conferring effectiveness against cerebral amyloid angiopathy [112].

## 6. Conclusion and Perspectives

The BBB accomplishes a paramount role in the maintaining of health and function of the brain; however, it also is a challenging hurdle for the treatment of brain disorders. For example, many drugs against cancers and neurodegenerative diseases have limited passage to the brain, and their local administration via surgical procedures is a tremendously invasive and impractical option. Thus, a variety of nanoformulations to tackle this issue have been proposed in the last decade. The use of nanocarriers may permit the administration of prospective drugs with poor crossing across the BBB. These nanocarriers would allow reaching the brain, regardless of the physicochemical characteristics of the molecule delivered. 

In this regard, the utilization of chitosan-decorated nanocarriers to transport therapeutic compounds through the BBB is an attractive alternative for enhancing their bioavailability and efficacy. Numerous research groups have explored their usefulness for different brain diseases; their findings indicate that the benefits of chitosan coating in the transit of nanoparticles to the brain are promising. Furthermore, chitosan has the classification of adjuvant and immunostimulatory by innate and adaptive immune responses, although this response may vary depending on the biological organism. However, due to the multiple combinations of chitosan coating on nanoparticles and differences in their composition at the surface level, information on aspects of molecular interaction or in advanced stages of clinical trials is limited. Moreover, although the principles on which the technology is based are positive charges of the amino groups, positive zeta potential, and high bioadhesion, it is difficult to predict the transit route through the BBB because it is not a specific interaction mechanism. Thus, there is a vast opportunity area for extensive studies pursuing aspects related to cellular interactions with the BBB, cellular internalization, pharmacokinetics, and long-term stability.

Furthermore, the coating of nanoparticles with chitosan offers the possibility of the reformulation for all those nanocarriers that do not exhibit a high transit to the brain but have already demonstrated a high percentage of drug loading and drug stability. Virtually all nanoparticle systems are eligible for chitosan coating, although the addition method will depend on the chemical composition of the nanoparticle. For higher added value, it is possible to make prior modifications to the chitosan for coating and then confer also targeting capability to specific regions of the brain. 

Therefore, the chitosan coating tool will continue to prosper and will offer innumerable opportunities for designing of nanoparticle formulations targeting the treatment of chronic CNS conditions. In this context, the use of in vitro cell models of BBB will be enormously valuable to accelerate the search for suitable nanoformulations. Likewise, in vivo evaluations will allow knowing drug amounts that reach the brain, as well as safety and efficacy profiles, to choose candidate nanocarriers for subsequent proofs of concept.

## Figures and Tables

**Table 1:** Chitosan decorated nanosystems applied to brain and CNS disorders.

Brain Desease/CNS Disorder	Nanosytem		Size (nm)		PDI		Z-Potential (mV)		Model Drug	Drug Loading (%)	Chitosan Coating Conditions/Method	Additional Functionalization/Targeting Agents	Reference
	Type	Nature of Nanocore	Non-Decorated	Decorated	Non-Decorated	Decorated	Non-Decorated	Decorated					
Brain cancer (Glioblastoma, and tumor diagnosis)	Polymeric NPs	PLGA	117.35 ± 2.37	140.88 ± 5.15	−−	−−	−14.94 ± 4.05	10.33 ± 0.26	Curcumin	LE: 39.48 to 82.67	Adsorption by immersion in CS solution at 0.02, 0.04, 0.06 %w/v. Crosslinkers: NHS and EDC	Anti-ALDH to target BCSCs and Salic Acid as BBB permeator	[13]
		PLGA	121.0	178.0	0.120	0.200	−34.0	46.0	Carboplatin	EE: 35.5 and DL: 1.8	Adsorption by immersion in solution of conjugated CS in a range concentration 0.0008-0.2 %w/v	Folic acid	[75]
		PLGA	168	177	−−	−−	−6.2 ± 2.5	19.6 ± 4.8	Carmustine/O^6^-Benzylguanine	LE: 8.97	Adsorption by immersion in CS solution at 0.3 %w/v	None	[14]
		PCL	170 ± 0.1	196 to 218	0.07 ± 0.02	0.25 to 0.20	−20 ± 0.6	39 to 54	Docetaxel	EE: ≃76.0 ± 3.0	Coating with CS amounts at 0, 0.01 and 0.025 %w/v	None	[79]
		PCL	−−	168 to 185	−−	0.12 to 0.16	−−	28.95 to 33.8	Lipid-core with Simvastatin	EE: ≃100	Interfacial deposition of pre-formed CS, aqueous phase contained 0.1 %w/v low-MW CS and high-MW CS	None	[47]
	Polymeric Micelles	Pluronic P123/F68^®^	−−	51.5 ± 12.3	−−	0.692 ± 0.09	−−	22.38 ± 4.15	Myricetin	EE: 91.72 ± 12.68 and DL: 15.63 ± 1.82	By adding an aqueous solution of CS (40 mg/20 mL) and incubation at 75 °C and dialysis against de-ionized water for 24 h	None	[80]
		D-**α**-tocopheryl glycol succinate 1000 (TPGS)	13.89 ± 1.2	14.25 ± 2.9	0.22 ± 0.05	0.37 ± 0.03	−4.91 ± 0.58	−2.32 ± 0.05	Docetaxel	EE: 98.8 ± 1.9	TPGS-COOH activated was conjugated to the CS by EDC and NHS in phosphate buffer saline (pH 5.5)	Transferrin	[81]
	Lipid NPs	SLN (Behenic Acid)	299.2 to 386.4	374.5 to 624.3	0.053 to 0.167	0.196 to 0.227	−3.04 to −3.41	15.46 to 23.79	Paclitaxel (PTX)	EE: 24 to 83 and DL: 28 to 100 gPTX/mg	Glycol chitosan was added at the beginning of the preparation of SLN where micellar solution was formed	None	[82]
		SLN (Vitamin E and Gelucire 44/14^®^)	−−	117 to 203	−−	0.131 to 0.216	−−	−−	Temozolomide	EE: 71.33 to 88.45 and DL: 7.11 to 9.23	NPs were converted to hydrogel using CS at 1.0 %w/v solution, adding slowly with constant stirring for half an hour	None	[86]
	Inorganic NPs	Iron Oxide	7.5 ± 1.3	76 ± 4	−−	0.16	−−	4 ± 7.4	O^6^-Benzylguanine	DL: 150 ± 14 BG molecules/NP	Via a coprecipitation method. CS-grafted-PEG (150 mg) was mixed with iron chlorides (9 mg Fe^2+^, 15 mg Fe^3+^) in 2.18 mL of degassed DI water until complete nucleation of NPs	Tumor targeting peptide, Chlorotoxin	[57]
			34.2 ± 5.4	57.1 ± 1.4	−−	−−	17.75 ± 0.64	18.63 ± 1.27	DNA encoding Human tumor necrosis factor α-related apoptosis-inducing ligand (TRAIL)	Effcient DNA binding	By reaction with aminated poly(ethylene glycol) residues of CS–PEG and CS–PEG–PEI copolymers	Chlorotoxin	[55]
			7	33	−−	−−	−−	4.2	−−	−−	NPs were synthesized in the presence of CS-graftedPEG via coprecipitation of ferrous and ferric chlorides with ammonium hydroxide.	Chlorotoxin and Cy5.5 (near-IR fluorophore)	[98]
			7.5	111.9 ± 52.4	−−	−−	−−	19.6 ± 5.7	siRNA to knockdown green fluorescence protein (GFP) expression	DL: 3.8 siRNA molecules/NP	nanoparticles were coprecipitated in the presence of chitosan-grafted PEG polymer	Chlorotoxin	[56]
			8	67.2 to 71.2	0.203	0.204	−0.028	0.182	Anti-CD20 single chain variable fragment-streptavidin fusion protein	DL: 0.7 FP molecules per NP	Via a coprecipitation method. CS-grafted-PEG (150 mg) was mixed with iron chlorides (9 mg Fe2+, 15 mg Fe3+) in 2.18 mL of degassed DI water until complete nucleation of NPs	Oregon Green 488	[96]
Alzheimer’s Disease	Polymeric NPs	PLGA	136.2 ± 1.09	142.3 ± 2.57	0.093 ± 0.005	0.091 ± 0.09	−27.29 ± 0.97	46.6 ± 1.87	Lutein	EE: 83.97 ± 1.03 and DL: 3.95 ± 0.03	By electrostatic interaction of CS at 0.01, 0.02, 0.03 and 0.04 %w/v	None	[15]
		PLGA	78.1 ± 3.7	125.4 ± 9.1	0.182 ± 0.027	0.197 ± 0.025	−21.2 ± 0.8	36.3 ± 4.0	Huperzine A	EE: 77.0 ± 3.9	By reaction with Mal-TMC (3 mg/7.5 mL)	Lactoferrin	[16]
		PLGA	−−	≈191.0 ± 5.0	−−	−−	−−	≈23.0 ± 3.0	Rosmarinic Acid	EE: 50.0 ± 2.0 (max)	Crosslink of PAAM-CS at 0.05 %w/v with EDC and NHS	Material 197 and ApoE	[76]
		PLGA	217.33 ± 6.82	267.67 ± 2.52	−−	−−	−7.64 ± 0.74	32.02 ± 2.65	6-Coumarin Probe	EE: 84.24 ± 4.22	Redispersion of NP in CS solution at 0.3, 0.5, and 0.7 %w/v by sonication and mix	Anti-Aβ antibody	[77]
	Lipid NPs	SLN (Witepsol E 85^®^)	335.76 ± 34.81	358.44 ± 25.89	0.013 ± 0.00	0.028 ± 0.02	−17.31 ± 0.68	10.54 ± 0.75	BACE1 siRNA	−−	A CS solution at 1 %w/v is added at 1:1 w/w NP suspension, magnetically stirred overnight	Rabies virus glycoprotein known as RVG-9R	[83]
	Nanovesicles	Niosomes (Tween-20^®^)	165.2 ± 3.1	180.2 ± 1.5	0.211 ± 0.020	0.248 ± 0.016	−41.6 ± 1.4	29.5 ± 1.6	Pentamidine	EE: 10	By adding CS (0.05 mg/mL in acetate buffer 0.2 M, pH 4.4) solution to the different niosome samples (1:1 ratio), stirring for 1 h at room temperature	None	[94]
Parkinson’s Disease	Lipid NPs	NLC (Precirol ATO5^®^ and Mygliol^®^)	201.5 ± 5.6	205.9 ± 6.3	0.315 ± 0.03	0.275 ± 0.02	19.9 ± 3.1	21.9 ± 1.8	Glial cell-derived neurotrophic factor (GDNF)	EE: 87.62 to 87.66 and DL: 1.31 μgGDNF/mgNP	TAT was covalently linked to CS, then NLC dispersion was added dropwise to the TAT-CS solution under continuous agitation for 20 min at room temperature	Cell-penetrating peptides; transactivator of transcription (TAT)	[49]
		NLC (Flaxseed oil and Tristearin)	38.41 ± 2.23	44.45 ± 1.5	0.309 ± 0.02	0.281 ± 0.05	−11.4 ± 0.98	16.15 ± 0.9	Ropinirole-dextran sulphate nanoplex	EE: 92.75 ± 2.30 and DL: 17.26 ± 1.10	0.5% w/v of aqueous TMC-CS solution was added to the aqueous dispersion of NLC, stirring for 2 h	None	[87]
	Liposomes	Phosphatidylcholine	−−	−−	−−	−−	−−	−−	Levodopa	−−	Electrostatic adsorption using a CS solution	None	[92]
	Emulsions	Microemulsion (Capmul MCM L8^®^, Tween-80^®^, PEG400 or Transcutol^®^)	24.9 ± 4.60	37.1 ± 8.80	−−	−−	−6.82 ± 2.80	13.7 ± 2.90	Cabergoline	Load of 0.167 %w/w	By adding CS solution (1 %w/w in acetate buffer pH 5) with stirring to the continuous phase such that the final content of chitosan in the formulations is 0.5 %w/w	None	[102]
Epilepsy	Polymeric NPs	PLGA	93.46 ± 3.94	106.31 to 142.43	0.106 ± 0.01	0.239 to 0.364	−12.63 ± 0.08	21.64 to 24.34	Catechin Hydrate	EE: 80.36 to 81.66 and DL: 5.98 to 6.87	Immersion in acidic (0.50% of acetic acid) CS solution (2.0 or 4.0 mg/mL) with 2.0 h of interaction	None	[17]
		PLGA	85.12 to 89.3	91.9 to 96.5	0.314 to 0.332	0.111 to 0.255	−2.53 to −3.47	17.47 to 20.29	Analogues of thyrotropin releasing hormone (NP-355 and NP-647)	EE: 47.94 to 52.56 and DL: 51.5 to 188.01 μg/mg of NPs	Electrostatic adsorption in CS solution at 1 mg/mL followed by stirring for 2 h at 400 rpm at room temperature	None	[18]
Cerebral Ischaemia	Polymeric NPs	PCL	163.4 to 234.6	201.3 to 283.6	0.146 to 0.364	0.253 to 0.409	−21.22 to −6.22	17.8 to 25.9	Glycyrrhizic Acid	EE: 77.94 to 74.43 and DL: 4.17 to 4.84	By incubation (2 h) with drug of an equal volume of CS solution (2 mg/mL in 65% acetic acid)	None	[20]
		PCL	181.3 to 254.0	224.5 to 284.0	0.143 to 0.297	0.216 to 0.419	− 22.31 to − 28.17	18.64 to 26.04	Eugenol	EE: 68.13 to 71.04 and DL: 4.29 to 5.14			[21]
	Emulsions	Nanoemulsion (Capmul MCM^®^, Tween-80^®^ and PEG-400)	91.39 ± 1.89	98.31 ± 1.17	0.372 ± 0.014	0.386 ± 0.021	−19.24	13.91	Naringenin	Load of 2.0 %w/v	At the end of emulsion formation, CS Solution is added (2.3 mL at 0.50 %w/v)	None	[100]
Schizophrenia and Bipolar Disorders	Polymeric NPs	PLGA	−−	306.1 to 700.0	−−	0.173 to 0.462	−−	5.67 to 24.7	Chlorpromazine Hydrochloride	EE: 18.61 to 36.72 and DL: 2.32 to 4.59	Via amide bond formation mediated by carbodiimide, with 12 h immersion at room temperature	None	[19]
	Lipid NPs	NLC (Glyceryl monostearate and Oleic Acid)	167.30 ± 7.52	181.58 to 186.97	−−	−−	−4.34 ± 1.37	5.51 to 18.88	Asenapine Maleate	EE: 82.46 to 84.24	Glycol CS solutions at 0.01, 0.05, 0.1, 0.2 and 0.4 %w/v were added to NCL suspension, stirring for 24 h	None	[88]
	Emulsions	Nanoemulsion: Capmul MCM^®^, Tween-80^®^ and polyethylene glycol 400)	20.1 ± 1.65	23.6 ± 2.11	0.264 ± 0.08	0.292 ± 0.06	−28.41 ± 2.14	−25.5 ± 1.32	Olanzapine	Load of 8.5 mg/mL (Drug Content of 97.96 0.24%)	By addition of CS (0.50 %w/w) to aqueous phase in emulsion preparation. The dispersion was stirred for 1 h	None	[101]
Migraine and pain	Lipid NPs	SLN (Glycerol Tripalmitate)	−−	192.0 to 301.4	−−	−−	−−	30.2 to 51.4	Sumatriptan Succinate	EE: 76.3 to 91.1	CS solution (1% glacial acetic acid) as aqueous phase (with 150 a 250 mg of CS) is incorporated in the solvent injection method	None	[84]
		NLC (Compritol^®^ and Labrafil^®^)	−−	255	−−	0.27	−−	34.1	Almotriptan Maleate	EE: 80	Adsorption by immersion in CS solution	None	[89]
	Nanovesicles	Bolaamphiphilic vesicles (bolalipids GLH-19 and GLH-20)	67.8 to 117.2	−−	0.155 to 0.277	−−	39.5 to 53.0	−−	Kyotorphin and Leu-Enkephalin (analgesic peptides)	EE: 5.8 to 11.2	CS-Vernolic acid conjugate was added at a molar concentration 5-fold lower than GLH-19 or GLH-20, at a molar ratio of 2:1, during film formation	None	[95]
	Polymeric NPs	PLA	121.2 ± 5.2	140.5 ± 5.4	−−	−−	−29.28 ± 2.39	33.71 ± 3.24	Neurotoxin from venom of Naja naja atra	EE: 75.17 to 83.51	Addition of CS solution (0.2 %w/v) in second aqueous phase in the resulting w/o/w emulsion, sonicated for 53 s	None	[78]
Neuroprotection	Polymeric NPs	PLGA	99.6 ± 6.3	146.7 ± 5.1	−−	−−	−18.3 ± 1.2	21.0 ± 2.9	Coenzyme Q10	DL: 8.8	By covalently coupled of TMC via a carbodiimide-mediated link	None	[59]
	Lipid NPs	SLN (Palmitic Acid)	138.8 ± 7.6	311.9 to 412.0	0.15 ± 0.04	0.24 to 0.26	−29.67 ± 1.20	27.08 to 35.70	Curcumin	EE: 93.12 ± 0.06 and LC: 4.04 ± 0.01	Surface modified with TMC by charge interaction (50:1-CS:SLN w/w) by dispersion in distilled water and stirring for 10 h	None	[85]
		NLC (Precirol ATO5^®^ or Dynasan 114^®^ and Miglyol^®^)	107.12 and 159.35	114.48 and 191.89	0.342 and 0.361	0.287 and 0.386	−30.30 and − 19.12	28.40 and 41.50	Neurotrophic factor human insulin-like growthfactor-I	EE: 90.28 ± 0.4	NCL dispersion is added dropwise to an equal volume of a CS solution (0.5 %w/v) kept under continuous agitation at room temperature for 20 min	None	[90]
	Lipid microparticles	Stearic Acid	68.5 ± 3.1 μm	76.3 and 84.5 μm	−−	−−	−12.7 ± 2.1	24.0 and 44.6	Resveratrol	EE: 76.5 and 81.0	Microparticles were added to CS solution (1.75 and 8.75 %w/v), during the cooling phase of emulsion method	None	[91]
Cerebrovascular Inflammation	Inorganic NPs	Gadolinium-Magnevist^®^ (MRI contrast agent)	164 ± 1.2	239 ± 4.1	−−	−−	11.9 ± 0.5	21.6 ± 1.7	Cyclophosphamide	DL: 21.7 ± 1.3	By emulsion-droplet coalescence technique developed at CS polymer concentration of 2.5 %w/v	Anti-amyloid antibody, IgG4.1	[99]
Others (Cachexia, Traumatic Brain Injury)	Liposomes	Lipoid S100^®^	147.3 ± 4.3	194 ± 6.1	0.119	0.198	−0.6 ± 0.3	6 ± 0.4	Ghrelin	EE: 9.8 ± 3.7	N-([2-hydroxy-3-trimethylammonium]propyl) CS chloride at 1 mg/mL is added dropwise to LP suspension under magnetic stirring at 3,000 rpm	None	[93]
	Inorganic NPs	Iron Oxide	7.0 ± 0.6 (22.7)	50 to 70	−−	−−	−−	−−	Reporter DNA (pCMV-td Tomato plasmid)	Efficient plasmid complex	CS-PEI magnetic-micelles (CPMMs) were prepared in a weight 1:1 ratio of CS-PEI (polyethyleneimine) (2 mg/mL)	MRI: Gadolinium chelates	[97]

ALDH = Anti-aldehyde dehydrogenase, ApoE = Apolipoprotein E, BBB = Blood-Brain Barrier, BCSCs = Brain Cancer Stem Cells, CS = Chitosan, DL = Drug Loading, EDC = 1-(3-dimethylaminopropyl)-3-3ethylcarbodiimide hydrochloride, EE = Entrapment or Encapsulation Efficiency, LC = Loading Capacity, LE = Loading Efficiency, MW = Molecular Weight, NCs = Nanocapsules, NHS = Hydroxysuccinimide, NLC = Nanostructured Lipid Carrier, NP or NPs = Nanoparticles, PAAM = Polyacrylamide, PCL = poly-𝛆-caprolactone, PLA = Poly (lactic acid), PLGA = Poly(lactic-co-glycolic acid), SLN = Solid Lipid Nanoparticle, TMC = N-Trimethylated chitosan.
